# A novel in vivo method to evaluate trueness of digital impressions

**DOI:** 10.1186/s12903-018-0580-9

**Published:** 2018-07-03

**Authors:** Emad A. Albdour, Eman Shaheen, Myrthel Vranckx, Francesco Guido Mangano, Constantinus Politis, Reinhilde Jacobs

**Affiliations:** 1 OMFS IMPATH research group, Department of Imaging and Pathology, Faculty of Medicine, University of Leuven and Oral & Maxillofacial Surgery, University Hospitals Leuven, Leuven, Belgium; 2 Department of Prosthodontics, Royal Medical Services, Jordanian Armed Forces, Amman, Jordan; 3 Department of Medicine and Surgery, Dental School, University of Varese, Varese, Italy; 4 Department of Dental Medicine, Karolinska Institutet, Stockholm, Sweden

**Keywords:** Intraoral scanners, Digital impression, Conventional impression, Trueness

## Abstract

**Background:**

Intraoral scanners are devices for capturing digital impressions in dentistry. Until now, several in vitro studies have assessed the trueness of digital impressions, but in vivo studies are missing. Therefore, the purpose of this study was to introduce a new method to assess trueness of intraoral scanners and digital impressions in an in vivo clinical set-up.

**Methods:**

A digital impression using an intraoral scanner (Trios® 3 Cart wired, 3Shape, Copenhagen, Denmark) and a conventional alginate impression (Cavex Impressional®, Cavex, Haarlem, the Netherlands) as clinical reference were made for two patients assigned for full mouth extraction. A total of 30 teeth were collected upon surgery after impressions making. The gypsum model created from conventional impression and extracted teeth were then scanned in a lab scanner (Activity 885®, SmartOptics, Bochum, Germany). Digital model of the intraoral scanner (DM), digital model of the conventional gypsum cast (CM) and those of the extracted natural teeth (NT) were imported to a reverse engineering software (3-matic®, Materialise, Leuven, Belgium) in which the three models were registered then DM and CM were compared to their corresponding teeth in NT by distance map calculations.

**Results:**

DM had statistically insignificant better trueness when compared to CM for total dataset (*p* = 0.15), statistically insignificant better trueness for CM when mandibular arches analyzed alone (*p* = 0.56), while a significantly better DM trueness (*p* = 0.013) was found when only maxillary arches were compared.

**Conclusions:**

Our results show that digital impression technique is clinically as good as or better than the current reference standard for study models of orthognathic surgery patients.

## Background

Conventional impression taking for dental cast preparation is still the clinical reference standard for replicating the intraoral situation [1]. Yet, such conventional approaches are considered cumbersome, bearing in mind the obstacles and challenges for both patient and dentist, including discomfort, nausea, unsatisfactory taste, time consumption, remakes in case of air bubble inclusion, forceful removal of highly retentive impressions with a risk for potential damage [2].

To overcome the drawbacks of conventional methods in dentistry, digital virtual models were introduced by Computer Aided Design/Computer Aided Manufacturing (CAD/CAM) solutions [3, 4]. Digital models can be created by indirect or direct approaches where indirect method uses laser optical scanning or computed tomography imaging of conventional impressions or plaster cast to produce digital virtual models [5, 6]. The direct method uses an intraoral scanning device to capture the patient dentition directly to produce a digital model that can be used to create temporary or final restorations [7–9].

During the last decade the use of intraoral digital impression systems have been steadily increasing. The possibilities and potential of digital impression taking as compared to the conventional approach may be related to its three dimensional representation on the computer, enable its versatile use for diagnostic model fabrication and integrated treatment planning. For clinical use, it is important to gain some idea on time and cost efficiency. These factors were assessed in some studies with a rather promising outcome [1, 2]. Another point is the system accuracy. Accuracy assessment has been targeted by several studies [10–13]. Yet, it is important to mention that accuracy is most often determined by standardized quality control measures using an in vitro set-up for assessment of precision and trueness. Precision expresses the closeness of repeated measurements to each other. Trueness describes the deviation of the measurement from the dimensions of a reference object. A higher precision means a more predictable measurement, while high trueness means less deviation from the reference object dimensions [9]. So far most accuracy studies mention low error levels, obtained via in vitro methodological set-ups. However, it should be indicated that all those studies start from laboratory testing or an in vitro model approach. A crucial point may however be the performance and accuracy in the clinic, as compared to the reference standard being conventional impression taking. There are a number of potential advantages, favouring digital impression taking to be implemented in daily dental practice. It is therefore crucial to also obtain information on the accuracy of such systems during in vivo use. It is indeed important to evaluate precision and trueness in a clinical environment in the presence of the patient, the operator and related factors that might affect accuracy such as blood and saliva in the mouth, patient movement, operator movement, obstructions by cheek and tongue, reflection of light by intraoral structures and restrictions of space inside the patient’s mouth [14]. Studying precision in the clinical set up can be done by repeating the scan of the same dentition intraorally multiple times and measure the deviations among these impressions [12, 15]. Assessing clinical trueness is more challenging, since the dimensions of the natural dental structure for which the digital or conventional impressions are made need to be captured accurately to be used as a reference model for comparison [16].

Focusing on study models made for orthognathic surgery patients, the aim of this study was to introduce a new method to validate the trueness of digital impressions of teeth scanned with an intraoral scanner and conventional impression (clinical standard) when compared to the corresponding natural teeth after extraction and scanned with a high resolution scanner (gold standard) in an in vivo clinical set-up.

## Methods

### Patient selection

Two patients who required full teeth extraction from Oral and Maxillofacial Surgery department (University Hospitals of Leuven, Belgium) were included in this study which was approved by the Ethical Review Board of the University Hospitals Leuven (S55619 ML9535, University Hospitals Leuven), signed informed consents were obtained from participants.

### Impressions and collecting teeth protocols

Before the surgical procedure, the upper and lower dentitions of each patient were digitally captured in the dental office with a chair-side intraoral scanner (Trios® 3 Cart wired, 3Shape, Copenhagen, Denmark) one time by a single experienced operator (first author) according to the manufacturer instructions for full arch scanning. For the mandible, one continuous motion starting on occlusal surfaces of posterior teeth starting from one side to the other with alternating movement on the anterior buccolingual area. Followed by scanning the lingual surfaces of all teeth from one side to the other. Finally rolling to buccal side and scan buccal surfaces of all teeth. For the maxilla, the same procedure was repeated except scanning the buccal surfaces of teeth before palatal ones and ending with scanning the palate.. A digital model (DM) was created and exported in stereolithographic (STL) format for each jaw.

One upper and one lower Conventional alginate (Cavex impressional®, Cavex, Holland) impressions in best fitting trays were made for each patient by the same operator and sent to the dental lab to create plaster casts. These plaster casts were digitized via high resolution optical scanner (Activity 885®, SmartOptics, Bochum, Germany), with an accuracy of 4 μm as provided by the manufacturer. Data was exported in STL format and referred to as conventional model (CM).

**Fig. 1:**
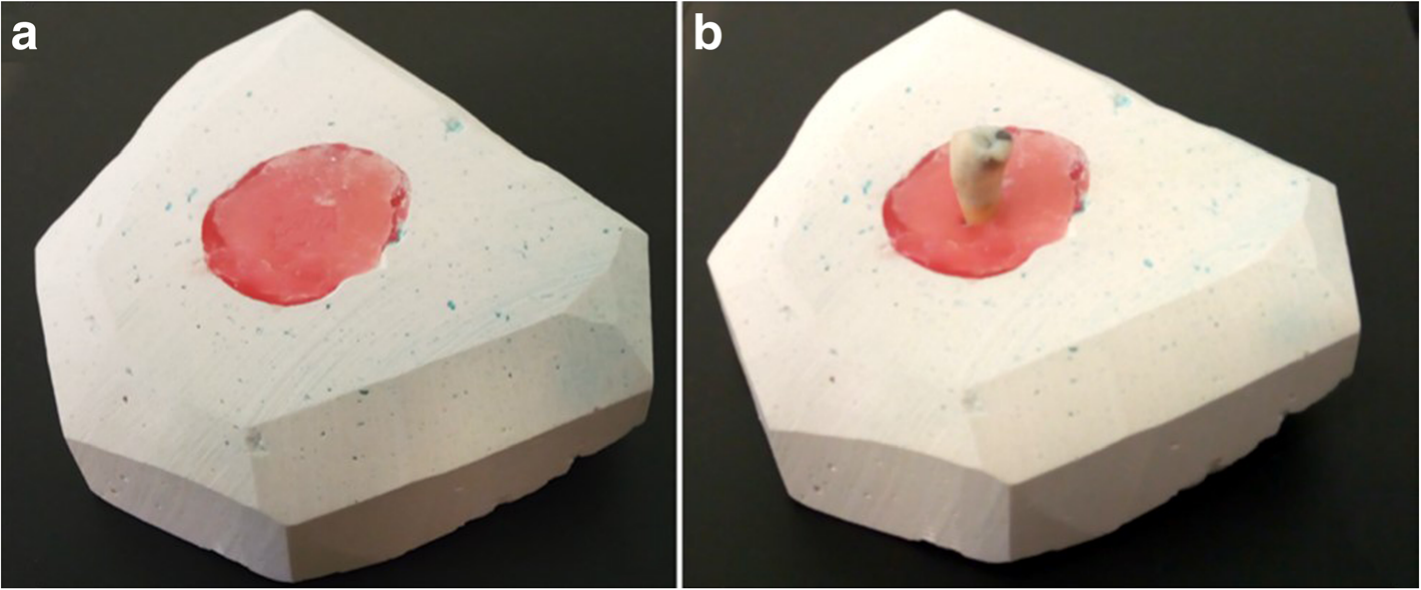
**a** Custom made gypsum base used to fix the teeth during scanning. **b** Each tooth was fixed by inserting its root into wax to keep the crown clear for scanning

During surgery, 30 extracted teeth which had full anatomic crowns or minor defects not affecting the dimensions of the crowns were collected from the patients to be used as reference models. All teeth with major defects or loose were excluded. After cleaning from blood and soft tissue residues, each tooth crown was scanned separately by the same lab scanner Activity 885^®^. Each tooth was fixed during scanning using a custom made gypsum base with a hole in the middle filled with modeling wax (Fig. 1a) by inserting the root into the wax and keeping the crown clear for scanning (Fig. 1b)***.*** Data were exported as natural teeth (NT) in STL format.

### Evaluation protocol

**Fig. 2:**
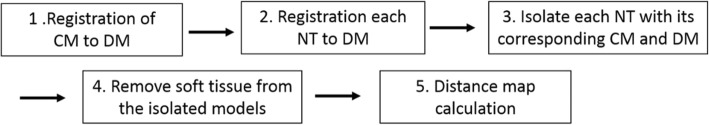
Evaluation protocol flaw chart

All the STL files of the DM, CM and NT for each patient were imported into a reverse engineering software (3-matic®, Materialise, Leuven, Belgium) to evaluate the trueness of DM and CM to its corresponding NT model. The steps are summarized in the flow chart in Fig. 2 and described as follows:

1. Register CM on DM using surface based registration (best fit alignment method).
2. Register each NT model onto its corresponding DM using surface based registration.
3. Isolate each tooth group using cutting planes which are parallel to the teeth.
4. Remove soft tissue from isolated CM and DM models due to lack of soft tissue in NT and to guarantee matched equal borders.
5. Calculate the distance maps (Euclidean distances) between surfaces using unsigned part comparison in 3-matic software providing a color-coded map. These distance maps were calculated between CM - NT and DM - NT separately provided that NT was used as the reference gold standard model in both cases.

The Root Mean Square deviation (RMS) was used to quantify and report degree of conformity of CM and DM compared to NT. RMS is a frequently used measure of the individual difference between values of a model and the values observed from the original object being modelled. RMS aggregates these individual differences into a single measure of predictive power.

### Statistical analysis

Statistical analysis was done using a dedicated statistical software (MedCalc version 16.4®, Ostend, Belgium). Normality of distribution was tested by the Shapiro-Wilk Normality test for CM and DM (maxilla, mandible and both jaws). Non-parametric Wilcoxon matched pairs test was used to compare degree of trueness between CM and DM to NT for maxillary, mandibular and both jaws, level of significance was set at *P* < 0.05.

## Results

Table 1 shows median, inter quartile range (IQR), mean and standard deviation (STD) for RMS values for CM and DM when compared with NT.

**Table 1:** Descriptive statistics of RMS values for CM and DM compared to NT

Model	Arch	Teeth (N)	Median μm	Inter quartile range μm	Mean μm	Standard deviation μm
CM	Maxilla	14	151	70	154	45
	Mandible	16	121	45	120	45
	Total	30	130	68	133	45
DM	Maxilla	14	113	65	106	37
	Mandible	16	118	53	133	57
	Total	30	118	38	119	48

**Fig. 3:**
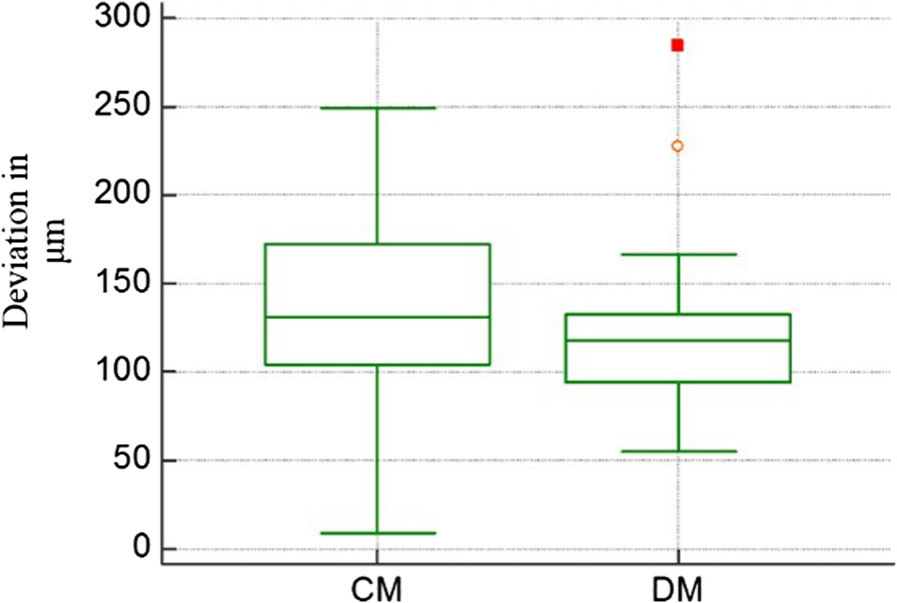
Boxplot of trueness deviations for CM and DM for total dataset (maxilla and mandible). The box represents the range of 50% of the difference measurements. The bar within the box represents the median trueness of CM and DM using the 25–75 percentile value. Square represents outlier difference measurements (more than 1.5 times the interquartile range). Circle represents extreme values (more than 3 times the interquartile range)

CM exhibited a total (maxilla and mandible) mean discrepancy and deviation of 133 ± 45 μm (range 10 -250 μm), while DM showed a total mean discrepancy of 119 ± 48 μm (range 60 -280 μm). Figure 3 shows a boxplot for deviations of CM and DM for both arches.

Results of Wilcoxon Matched Pairs Test for comparing CM and DM are reported in Table 2, statistically insignificant better trueness for DM compared to CM (*P* = 0.15) was found when total dataset was analyzed, statistically insignificant better trueness for CM when mandibular arches analyzed alone (*p* = 0.56). While a statistically significant difference was found between DM and CM for maxillary arches alone, favoring DM (*P* = 0.013).

**Table 2:** Trueness level of CM and DM to NT

Model	Arch	N(Teeth)	P-level
CM - DM	Maxilla	14	0.013*
	Mandible	16	0.56
	Total	30	0.15

*indicates significant difference (*P* < 0.05)

**Fig. 4:**
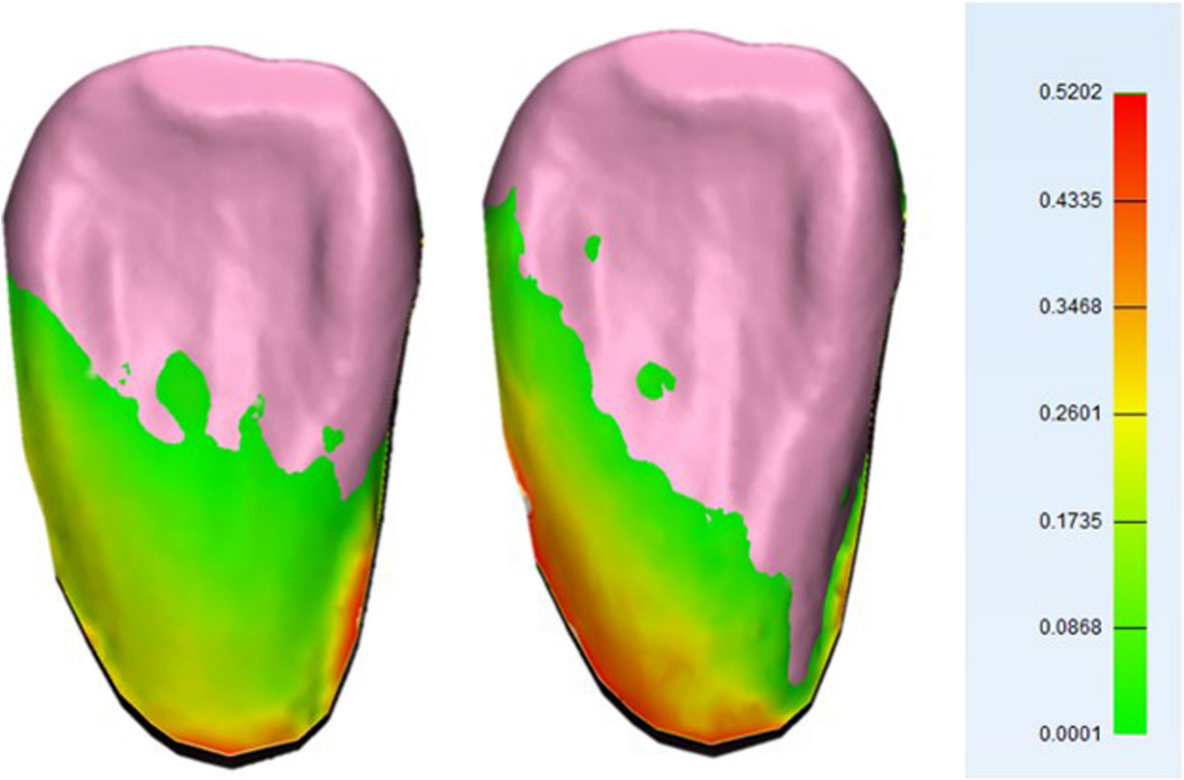
Color deviation map shows positive deviations in proximal and cervical areas. CM (left), DM (right). A positive value (red) in the color deviation map indicates that the CM and DM are larger than NT in these specific areas

**Fig. 5:**
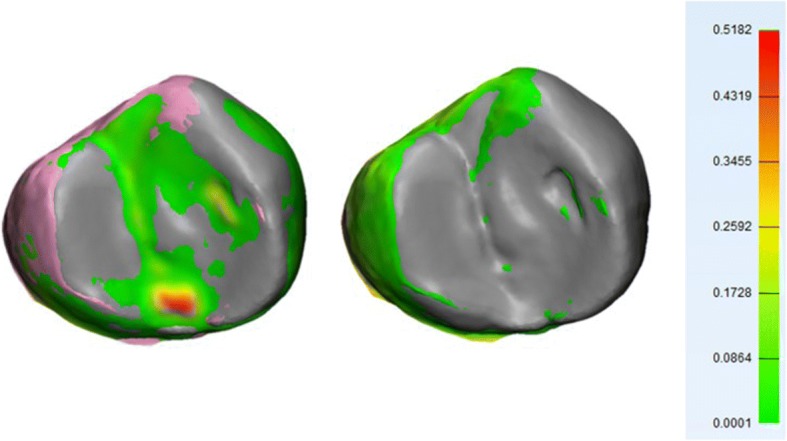
Positive localized deviations on the surface of CM (left) which are absent on the same surface in DM (right). A positive value (red) in the color deviation map indicates that CM is larger than NT in these specific areas

Visual assessment of color-coded deviation maps for teeth showed maximum positive deviations concentrated mainly in two areas: cervical and proximal regions in both CM and DM (Fig. 4), and occlusal surface only in CM (Fig. 5).

## Discussion

In this study, we presented a new in vivo method to evaluate the trueness of intraoral scanner by comparing conventional and digital impressions to the corresponding natural teeth after extraction.

DM using the former intraoral scanner showed better trueness values (119 μm) than CM (133 μm) when both arches were compared. Maxillary arches were significantly higher for DM than CM, while mandibular arches showed insignificant deviations between both methods with higher trueness for CM.

Cervical (buccal, lingual or proximal) deviation areas for CM can be caused by variable thickness or deficiency of impression materials between neighboring teeth, especially for alginate which has low tear resistance affecting the accuracy of gypsum model (Fig. 4 *left*) [2, 17–20]. While in DM, this can be caused by light reflection of saliva in interproximal areas as well as the difficulty of scanner light to penetrate these areas (Fig. 4 *right*) [21] affecting their accuracy in the mandible were saliva has higher concentrations. Another possible effect would be the proximity to gingival tissues which has different light reflective capabilities than tooth structure which might cause potential distortion of light transmitted by intraoral scanner in these areas. Anna et al. [22] found the same area to be affected, it was mentioned that best fit method used for comparison would cause an axial movement of the point-cloud for the model being compared, and such adjustment would cause positive distortions in the margin with reduced distortions at occlusal surface.

The second area with deviations was only shown in CM on the occlusal surfaces of posterior teeth and palatal (lingual) on anterior teeth. No specific pattern of distribution was detected. This might be attributed to unavoidable trapped air bubbles on the surface of alginate impression which are duplicated on gypsum cast surface and appear as local deviations (Fig. 5 *left*).

Less saliva content in upper arches, more flexibility and space for scanner tip movement and less movement of maxillary arches compared to mandibular ones could explain significantly better trueness for DM in maxillary arches.

Trueness of intraoral scanners has been examined in few studies, some by taking digital or conventional impressions for industrial manufactured models in controlled lab set-up [23], where the absence of in vivo set-up with its intraoral factors mentioned earlier might affect reported trueness results. Other studies used conventional and digital impressions taken for patients to be compared together using one of them as a reference [24]. These studies neglected the presence of inherited errors in this reference impression or the model created from it leading to less trueness and trusted results. On the other hand, there are studies that used marginal and internal fit of final crown and bridge restorations constructed using digital and conventional impressions to assess the trueness level [21]. In fact, this method involved the trueness of all steps in manufacturing final restoration, and not only the trueness level of impression itself [25].

Intraoral scanners have many advantages which include and not limited to better accuracy values as shown in our results, easy to use, patients’ comfort, physical storage space not needed, impressions can be sent directly to milling machine to finalize restorations in minutes, reduced labor work and fast with cost efficient implementation [26, 27], which encourage replacement of conventional impressions by digital ones for many cases of surgical planning and splints manufacturing in maxillofacial surgeries and orthodontic treatments.

In our study, all factors affecting trueness values were neutralized, up to the authors knowledge it was not mentioned in literature that digital and conventional impressions where made in vivo and compared to the same original intraoral structures captured in these impressions. Evaluation was limited to teeth as soft tissue could not be cropped with those teeth (NT) for comparison. Moreover, each tooth was separately evaluated as it is impossible to extract all the teeth and preserve their position accurately on a gypsum base as they are in the patient mouth to be examined for trueness as complete arch.

The use of only one intraoral scanner was another limitation of this study. However, this Trios scanner allows exporting STL files that are compatible with multiple CAD/CAM software. In addition, it supports a powder free, colored scans, ultrafast optical sectioning technique and confocal microscopy scanning technology [6, 28, 29]. Moreover, this study focused on the introduction of a new method to evaluate the accuracy of intraoral scanners in terms of trueness and not to compare several scanners.

Nevertheless the current results show that the digital impressions technique is clinically as good as or better than the current reference standard used for orthognathic surgery patients. Optimally clinical and digital impression taking for prosthetic work should yield error values of around 25 μm [30].

It is recommended to conduct more studies using other types of impression materials and commercial intraoral scanners and include more patient data to report on clinical accuracy rather than technical factory accuracy.

## Conclusions

Direct digital impression methods using intraoral scanning have many advantages over conventional impressions with accurate in vivo results. Yet the present clinical study indicates that the variability in scanner output is still large, with error levels somewhat around or below the conventional impression method.

## Abbreviations

CAD/CAM: Computer Aided Design/Computer Aided Manufacturing
CM: Conventional gypsum cast model
DM: Digital model of the intraoral scanner
IQR: Inter quartile range
NT: Natural extracted teeth model
RMS: Root Mean Square deviation
STD: Mean and standard deviation
STL: Standard Tessellation or Stereolithographic File
